# Identifying Site‐Level Data Integrity Risks in Alzheimer’s Trials via Subject‐Level Heuristics and a Novel Machine Learning Framework

**DOI:** 10.1002/alz70861_108662

**Published:** 2025-12-23

**Authors:** Joseph Geraci

**Affiliations:** ^1^ Department of Pathology and Molecular Medicine, Queen’s University, Kingston, ON, Canada; Arthur C. Clarke Center for Human Imagination, School of Physical Sciences, University of California, San Diego, La Jolla, CA, USA; NetraMark Corp, Toronto, ON Canada

## Abstract

**Background:**

Undetected data integrity issues in Alzheimer’s disease (AD) trials often appear as “paradoxical” participant profiles with atypical or implausible profiles that can distort efficacy signals and threaten the validity of study conclusions. A unique mathematically‐augmented machine learning (MAML) framework was used to identify high‐risk study sites based on patterns in subject‐level medical history data, despite a small sample size of trial sites.

**Methods:**

Using data from a trial evaluating treatment for mild to moderate AD, 1855 subject‐level variables were transformed into 7816 site‐level features by computing distributional summaries of subject medical data (min, mean, max) across 36 sites. Sponsor‐provided site‐risk labels were used to train classification models including large language models and several standard ML methods such as Random Forest and XGBoost (LLM/ML) in three scenarios:

(1) LLM/ML on all raw features

(2) LLM/ML on MAML‐derived features

(3) LLM/ML on MAML‐derived features and learned site relabeling.

Leave‐one‐out cross‐validation (LOOCV) assessed accuracy, sensitivity, specificity, and AUC due to the small sample size constraints.

**Results:**

Scenario 3 achieved the highest predictive performance:XGBoost yielded an AUC of 0.99 (accuracy 0.89, sensitivity 0.93, specificity 0.86). In contrast, Scenario 1 trained with all features peaked at an AUC of 0.25, underscoring the value of MAML’s data‐driven relabeling to uncover latent site‐level risks. The MAML framework grouped all five known high‐risk sites and nine additional sites into a high‐risk group defined by 10 key variables, predominately anxiety‐related. High‐risk sites consistently displayed statistical deviations from the canonical AD symptomatology structure.

**Conclusion:**

Pre‐randomization, baseline subject data, can effectively flag site integrity risks in AD clinical trials by detecting deviations from the standard spectrum of symptomologies via MAML. This deviation can be used to better evaluate clinical trial sites. Incorporating these insights into ongoing and future AD trials may enhance data quality, mitigate bias, and reinforce confidence in trial outcomes.

**Impact**: Due to AD’s clinical heterogeneity and overlap with neuropsychiatric symptoms, our scalable approach offers early detection of anomalous trial behavior, supporting more trustworthy efficacy assessments across AD and related psychiatric trials.

